# Crystallographic texture dependent bulk anisotropic elastic response of additively manufactured Ti6Al4V

**DOI:** 10.1038/s41598-020-80710-6

**Published:** 2021-01-12

**Authors:** Mangesh V. Pantawane, Teng Yang, Yuqi Jin, Sameehan S. Joshi, Sriswaroop Dasari, Abhishek Sharma, Arkadii Krokhin, Srivilliputhur G. Srinivasan, Rajarshi Banerjee, Arup Neogi, Narendra B. Dahotre

**Affiliations:** 1 Department of Materials Science and Engineering, University of North Texas, Denton, TX 76207 USA; 2 Department of Physics, University of North Texas, Denton, TX 76203 USA; 3 Center for Agile and Adaptive Additive Manufacturing, University of North Texas, Denton, TX 76207 USA

**Keywords:** Microscopy, Materials science, Structural materials, Metals and alloys

## Abstract

Rapid thermokinetics associated with laser-based additive manufacturing produces strong bulk crystallographic texture in the printed component. The present study identifies such a bulk texture effect on elastic anisotropy in laser powder bed fused Ti6Al4V by employing an effective bulk modulus elastography technique coupled with ultrasound shear wave velocity measurement at a frequency of 20 MHz inside the material. The combined technique identified significant attenuation of shear velocity from 3322 ± 20.12 to 3240 ± 21.01 m/s at 45$^\circ$ and 90$^\circ$ orientations of shear wave plane with respect to the build plane of printed block of Ti6Al4V. Correspondingly, the reduction in shear modulus from 48.46 ± 0.82 to 46.40 ± 0.88 GPa was obtained at these orientations. Such attenuation is rationalized based on the orientations of $\alpha ^\prime$ crystallographic variants within prior columnar $\beta$ grains in additively manufactured Ti6Al4V.

## Introduction

Although various additive manufacturing (AM) techniques associated with distinct physical phenomena have emerged, the laser powder bed fusion (LPBF) based AM is widely explored as it offers significant control over the geometric tolerance of the printed component^1–4^. Additionally, in LPBF-AM, a broad spectrum of process parameters allows monitoring the set of properties of the printed part^5–7^. LPBF-AM, by its principle nature, yields smaller melt pool dimensions ($\sim$ 30–100 $\upmu$m in depth and $\sim$ 80–400 $\upmu$m in width) in a significantly large volume of powder, which in turn leads to a rapid cooling rates (10$^5$–10$^7$ K/s) and steeper thermal gradients (10$^5$–10$^7$ K/m)^3,7^. Such rapid thermokinetics often result in microstructural anisotropy (texture) and heterogeneity, impacting the macroscopic physical properties^8^. These effects are mitigated by adjusting process parameters as well as by post-processing treatments. However, evaluating these aspects at every step is often carried out via X-ray diffraction, neutron diffraction, and electron backscattered diffraction (EBSD), where the sample preparation needs machining and polishing that can potentially modify the intrinsic structure produced during AM^9,10^. Moreover, the measurements via these techniques are limited to the surface/subsurface region and not representative of the entire component. Especially, these issues remain valid for evaluation of average static elastic constants that involves destructive and quasi-destructive testings^11^. Furthermore, the elastic constants evaluated using additively-printed tensile specimens may not represent the actual properties of the AM-component of different geometry when printed with the same set of process parameters. This is a likelihood as the spatial heterogeneity and anisotropy in the resultant microstructure are produced in the additively-printed tensile specimen. In addition, determination of the static elastic constants may not be a reliable choice for the components undergoing rapidly-varying and dynamic loading conditions during their applications.

Accounting the above-mentioned scenario, the non-destructive techniques (NDT) such as ultrasound technique is imperative to recognize bulk dynamic and anisotropic responses of the AM component. The ultrasound technique involves propagation of waves through the material to be evaluated either with longitudinal or transverse polarization to provide a non-destructive mechanical stimulus to the material whose response is reflected as a characteristic change of waves^12,13^. These responses include changing the attenuation coefficient, amplitude, and velocity of the ultrasonic wave, primarily due to scattering. These characteristics are influenced by the directionally varying crystallographic arrangements and the multidimensional defects, including vacancies, dislocations, coherent and incoherent phase/grain boundaries, and process-induced defects such as cracks and porosity^12^. So far, the bulk anisotropy has been explored using the ultrasound technique for single crystals, textured materials processed via thermomechanical treatments, and weldments^14–17^. Experimentally, the characteristics of ultrasound waves propagating through the material are monitored by varying the orientation of either longitudinal or shear ultrasound waves^16^. This technique is also useful in determining the grain size along with the crystallographic texture of the material, for which primarily the change in wave velocity, amplitude, and attenuation coefficient have been analyzed^17,18^. The use of ultrasound waves has been further evolved as the general convolution approach for validation of its capability to capture the bulk crystallographic texture information^19–21^. These studies have consistently reported qualitative comparison of ultrasound technique in line with EBSD and neutron diffraction outcome. Moreau et al.^22^ quantitatively indicated the accuracies within 2 degrees for the crystallographic orientation measured by ultrasound techniques when compared to EBSD. In the LPBF-AM technique, the thermokinetically-induced crystallographic texture is prominent, which also unveils various crystallographic traits pertaining to distinct directions. In light of this, the present paper focuses on the bulk crystallographic texture and associated elastic anisotropy effect in the laser powder bed fused additively manufactured (LPBF-AM) Ti6Al4V alloy. These efforts are accomplished with the aid of recently developed effective bulk modulus elastography (EBME) technique coupled with ultrasonic shear wave propagation in LPBF-AM Ti6Al4V alloy^23,24^. The investigation further correlates the effect of crystallographic variant on the microstructural morphology and the elastic anisotropy of LPBF-AM Ti6Al4V alloy.

## Methods and materials

**Figure 1:**
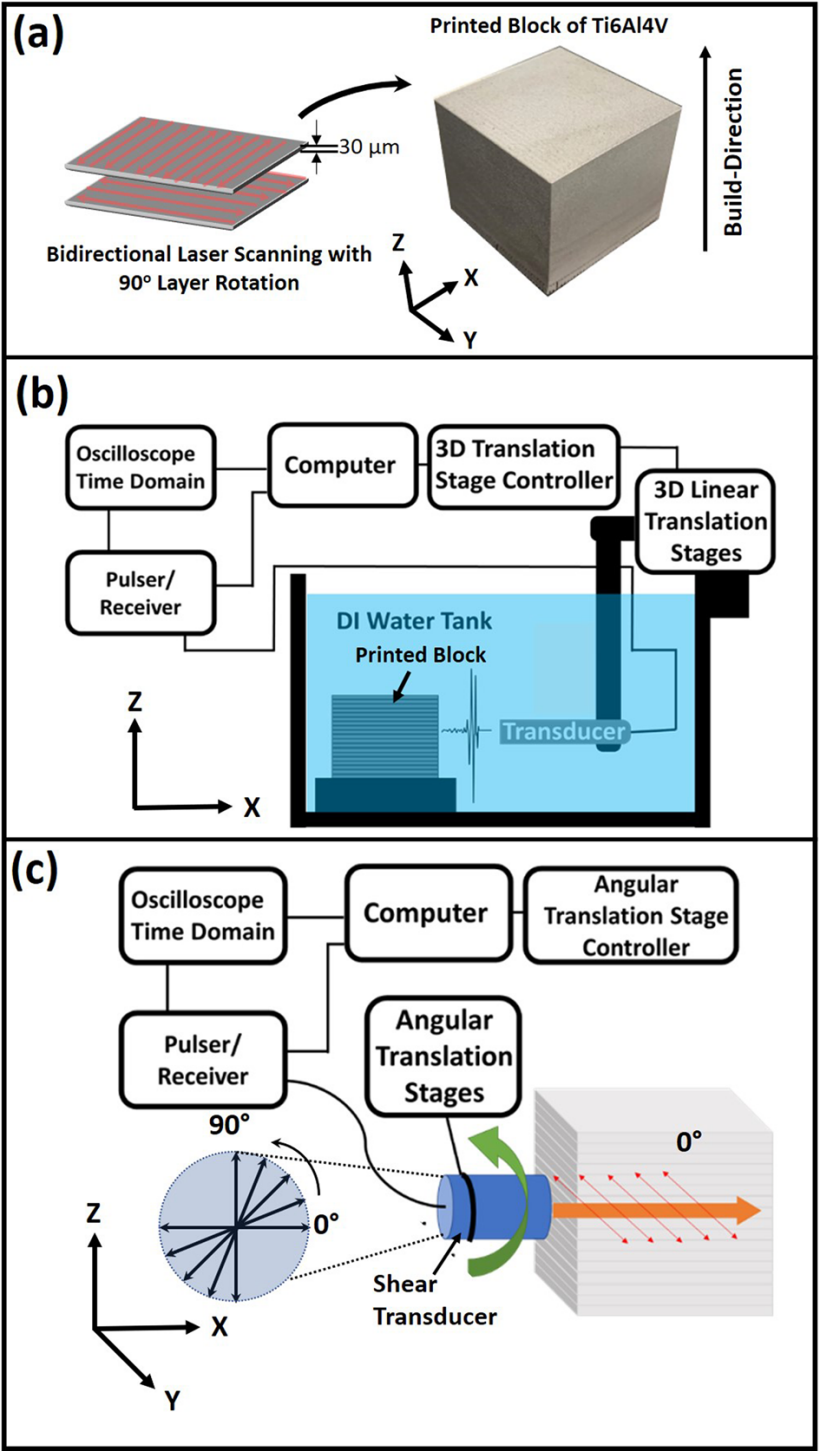
Set of schematics illustrating (**a**) the process of laser powder bed fusion, (**b**) setup for performing elastic bulk modulus elastography, and (**c**) experimental arrangement for measuring velocity of sound using shear waves.

LPBF printing of a cubical block (38 mm$^3$) of Ti6Al4V was carried out in AconityMIDI AM system ( a continuous wave Nd:YAG laser, Gaussian energy distribution, wavelength of 1070 nm, beam diameter of 85 $\upmu$m (D4$\sigma$)) using unimodal, and ultra-low interstitial grade 23, Ti6Al4V alloy powder (average particle size of 15–$45\, \upmu \hbox {m}$ and composition of Al5.5–6.5; V3.5–4.5; O $\le$ 0.13; C $\le$ 0.08; H $\le$ 0.012; Fe $\le$ 0.25; N $\le$ 0.05; and Ti Balance, all in weight %) obtained from Carpenter Technology. The build chamber was maintained under ultra pure Ar atmosphere ($\hbox {O}_2<50\, \hbox {ppm}$) throughout LPBF-AM process. The laser power and the scanning speed were kept constant at 150 W and 800 mm/s respectively. The laser scanning direction was reversed after each laser track (bidirectional) and this scan pattern was rotated by 90$^\circ$ between subsequent layers (Fig. 1a). The center to center distance (hatch spacing) between the adjacent laser tracks was 120 $\upmu$m. Combination of these laser processing parameters generated laser energy density of 52.08 J/mm$^3$ on the sample surface. Further details about the LPBF-AM process can be located in previous publication by the authors^3,4^. In order to conduct post LPBF-AM process, NDT and microstructural characterization/analysis, the AM printed Ti6Al4V cubical block was separated from the circular build plate using electrode discharge machining.

The elastic properties of LPBF-AM Ti6Al4V samples were analyzed in a non destructive manner by coupling two types ultrasonic techniques: 1. Effective Bulk Modulus Elastography (EBME) using longitudinal waves and 2. Shear wave propagation in the material (Fig. 1b,c). EBME was performed using a 20 MHz immersion based Olympus Panametrics V312 transducer of 3mm diameter (Fig. 1b) whereas shear wave velocity was measured using Olympus Panametrics V211 0.125-in. diameter 20 MHz defocused shear transducer (Fig. 1c). All the scans were performed on the cube faces (YZ and XZ planes) parallel to the LPBF build direction (Z). In case of EBME, a JSR Ultrasonic DPR 300 Pulse/ Receiver operated the pulse source and time trigger. The data was collected by a Tektronix MDO 3024b oscilloscope. The scanning rate was 512 signals per 20 seconds. On the other hand, shear wave velocity was scanned at multiple location (6 $\times$ 3 locations on YZ plane of the cube at an interval of 3.5 mm). At each location, polarized plane of shear wave was rotated around X-axis in the range of 0–90$^\circ$ with an interval of 22.5$^\circ$ with respect to the build plane (XY) as depicted in Fig. 1c. The angular resolution of the equipment was 0.5$^\circ$.

With EBME technique^23,24^, the average bulk density $\rho$ and dynamic bulk modulus $K_{d}$ were calculated at different locations of the cube face (YZ) by probing its acoustic impedance (Z) and using the classical speed of sound theory: $K_{d}$ = Zc and $\rho$ = Z/c, where c is the velocity of sound in the material (Fig. 1b). The ambient deionized water provides stable values of impedance while probing. The emitted pulse envelope by the transducer is separated into two echoes at the front and rear interfaces of the block due to an impedance contrast between the sample material and the deionized water. The sample acoustic impedance ($Z_1$) can be calculated by the known acoustic impedance $Z_0$ (Pa.s/m) of deionized water and the ratio of the second echo and the difference between the emitted wave with the first echo as given in Eq. (1)

1 $$\begin{aligned} \frac{Z_1}{Z_0}=\frac{-1-\alpha -\sqrt{4\alpha +1} }{\alpha -2} \end{aligned}$$

where $\alpha =\frac{P_1}{P_e-(Z_1-Z_0)|P_0|}$ is the experimental factor calculated using $P_e$, which is the sound pressure of the emission source, and $P_0$ and $P_1$ are experimentally measured sound pressures (Pa) of the first and second reflection from the front and rear surface of the block.

The temporal delay of the two reflected envelopes and the longitudinal speed of sound $C_L$ (m/s) allows calculating $K_d$ (Pa) and $\rho$ (kg/m$^3$) using Eqs. (2) and (3), respectively.

2 $$\begin{aligned} K_{d}= & {} C_L Z_0\;\frac{-1-\alpha -\sqrt{4\alpha +1} }{\alpha -2} \end{aligned}$$

3 $$\begin{aligned} \rho= & {} \frac{Z_0}{C_L}\;\frac{-1-\alpha -\sqrt{4\alpha +1} }{\alpha -2} \end{aligned}$$

With this bulk density $\rho$ (kg/m$^3$) and the shear wave velocity $C_T$ (m/s) measured at a given location with different orientations of polarized shear wave plane, the dynamic shear modulus can be obtained as follows:

4 $$\begin{aligned} G_{d}=\rho {C_T}^2 \end{aligned}$$

Similarly for comparison purpose, ultrasonic measurements were also carried out on the reference wrought Ti6Al4V plate. Here it should be noted that the as received wrought Ti6Al4V was in an annealed condition, and it possessed equiaxed grain texture.

To get insights into microstructure and crystallographic texture and interpret the data from ultrasonic measurements, the LPBF-AM samples were sectioned in XZ plane using an oil cooled slow speed diamond laced wheel saw. The sectioned samples were placed in epoxy mounts and prepared using protocol described in previous paper by the authors^3^. The microstructure was observed in FEI Nova NanoSEM 230 equipped with a Hikari super electron backscatter diffraction (EBSD) detector operated at 20 kV and spot size of 6. The sample was mounted on 70$^\circ$ pre-tilted holder and kept at a working distance of 12 mm. The EBSD analysis was performed using scanning step size of 0.5 $\upmu$m. The inverse pole figure maps and pole figures were generated from the EBSD data with the aid of TSL OIM Analysis 8.0 software. A numerical reconstruction of the parent phase structure and corresponding pole figure were determined by using MTEX (version 5.5), an opensource Matlab toolbox^25^. The reconstruction was mainly based on the Burgers orientation relationship between the parent and the product phase. Transmission Electron Microscopy (TEM) was carried out using 200kV FEI Tecnai G2 TF20. The sample preparation for TEM was done by using the FEI Nova 200 dual-beam focused ion beam (FIB).

**Figure 2:**
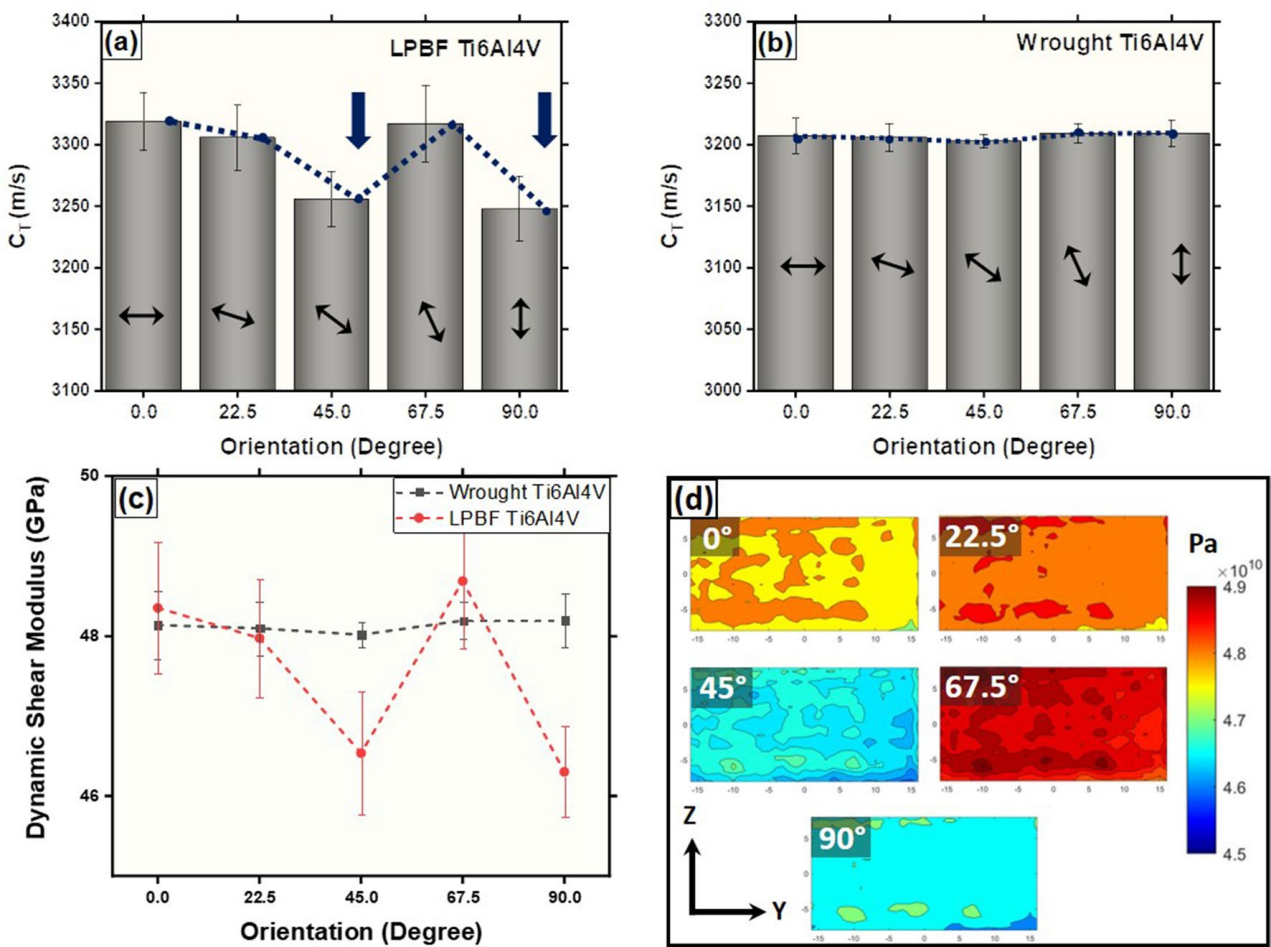
Variation in velocity of shear waves as a function of shear wave plane orientation with respect to build plane normal for (**a**) LPBF-AM Ti6Al4V and (**b**) wrought reference Ti6Al4V sample and (**c**) corresponding shear modulii of wrought and LPBF-AM Ti6Al4V. (**d**) Shear modulus contour maps on YZ plane of LPBF-AM Ti6Al4V cube at different shear wave plane orientations.

**Table 1:** Orientation dependent dynamic elastic moduli of LPBF-AM Ti6Al4V.

Orientation (°)	Dynamic Shear modulus (GPa)	Dynamic Youngs modulus (GPa)	Dynamic Poissons ratio	Dynamic Bulk modulus (GPa)
0	48.46 ± 0.82	124.96 ± 1.84	0.302 ± 0.002	102.44 ± 0.68
22.5	48.08 ± 0.74	124.71 ± 1.66	0.304 ± 0.001	102.44 ± 0.68
45	46.64 ± 0.76	120.84 ± 1.73	0.309 ± 0.001	102.44 ± 0.68
67.5	48.80 ± 0.98	123.99 ± 1.99	0.305 ± 0.003	102.44 ± 0.68
90	46.40 ± 0.88	120.31 ± 1.95	0.310 ± 0.002	102.44 ± 0.68

**Figure 3:**
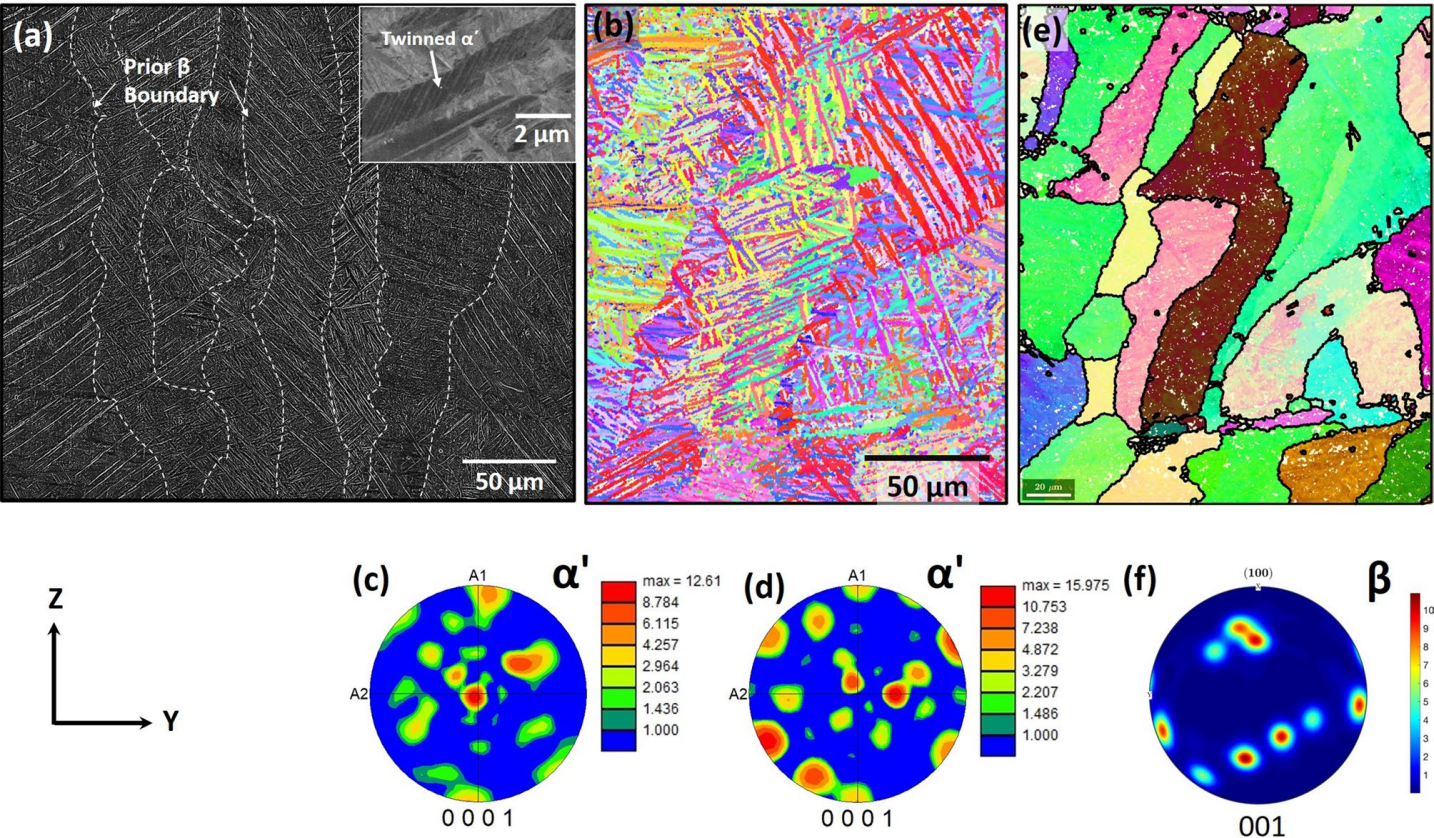
(**a**) SEM and (**b**) EBSD IPF map of LPBF-AM Ti6Al4V cube from YZ-plane and (**c**) corresponding and (**d**) additional 0001 pole figure texture plots from YZ-plane. (**e**) Numerically reconstructed prior $\beta$ inverse pole figure map using Mtex toolbox 5.5^25^ and (**f**) corresponding 001 pole figure.

**Figure 4:**
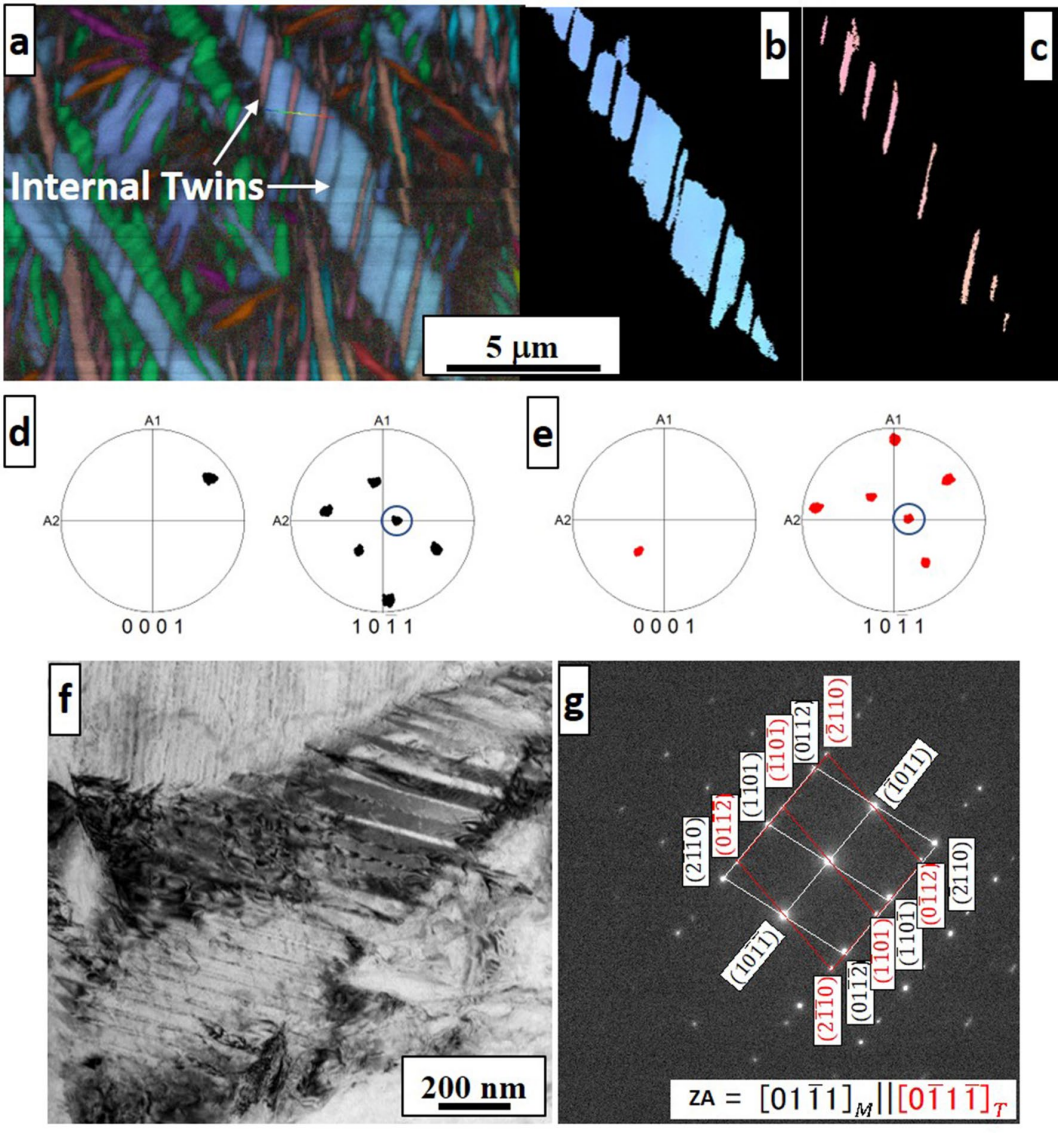
High resolution SEM-EBSD analysis of internally twinned martensite plates in the LPBF-AM Ti6Al4V sample. (**a**) Shows an inverse pole figure map with multiple internally twinned martensite plates. (**b**, **c**) Show IPF maps of a single plate with the matrix region and twinned region, respectively. Corresponding pole figures showing the {0001} and {10$\bar{1}$1} poles of the matrix region of the plate (**d**), and the twinned region of same plate (**e**), exhibit a common {10$\bar{1}$1} pole (marked with open circles). (**f**) Bright-field TEM image showing a high density of twins within a single martensite plate (**g**) Corresponding electron diffraction pattern recorded along a common [10$\bar{1}$1] type zone axis for both the matrix and the twin orientations.

**Figure 5:**
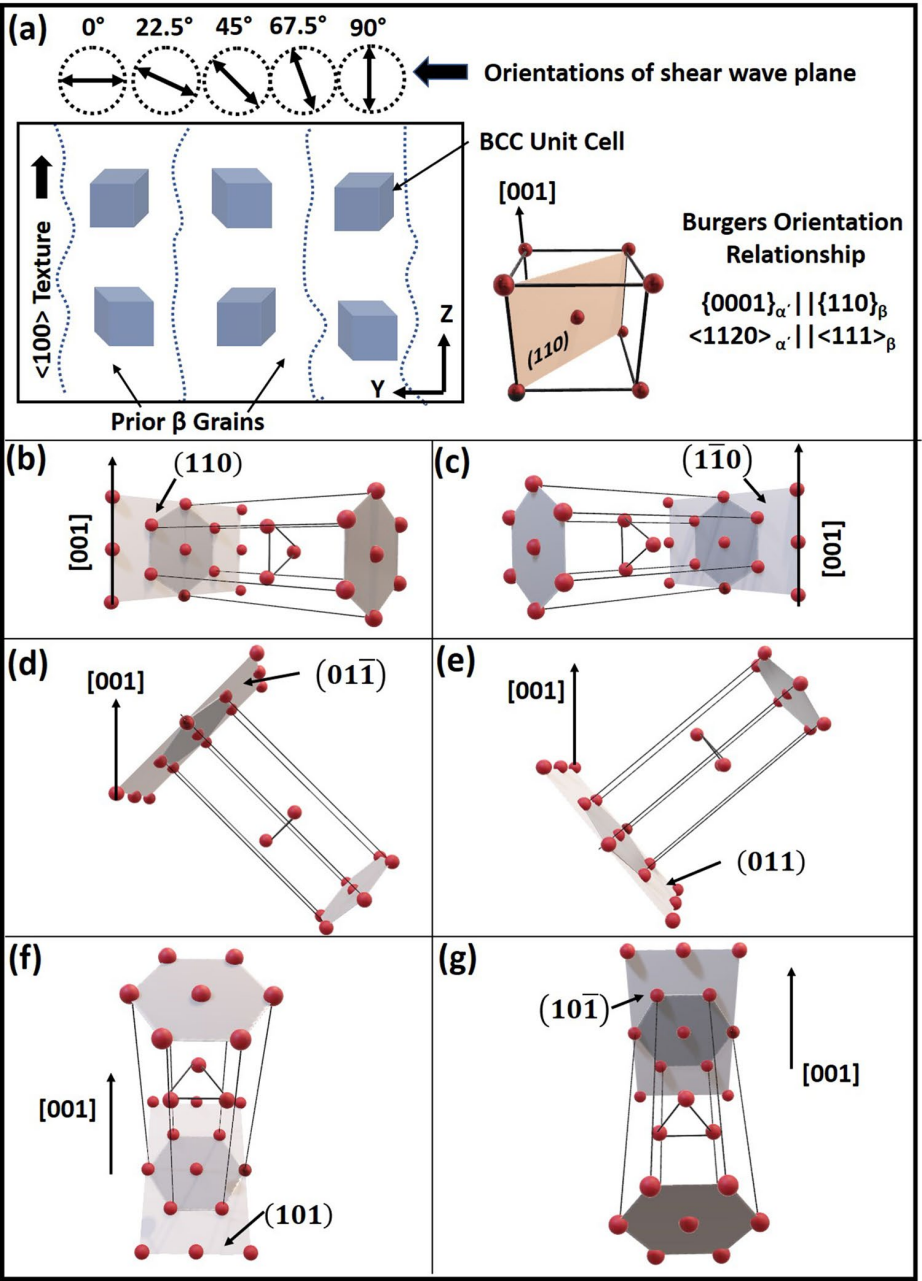
Schematics of (**a**) prior $\beta$ grains and (**b**) BCC unit cell with (**c**–**g**) possible variants of $\alpha ^\prime$ martensite HCP unit cell.

**Figure 6:**
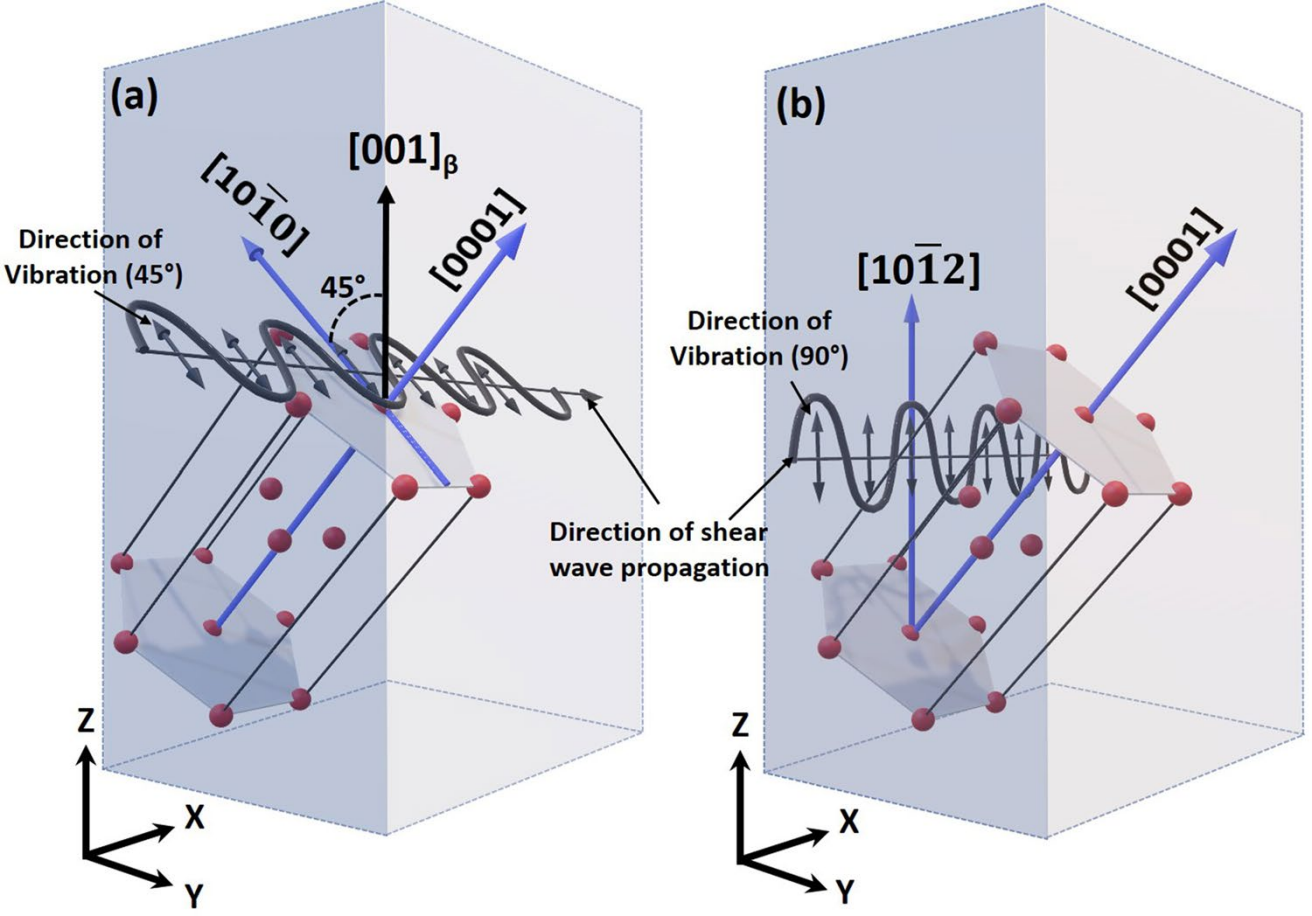
Schematic visualization of shear wave plane coinciding with the elastically soft direction at (**a**) 45$^\circ$, (**b**) 90$^\circ$ orientations.

## Results and discussion

The preliminary results on phase evolution in LPBF-AM Ti6Al4V examined using X-ray diffraction, transmission electron microscopy, and atom probe tomography have been reported in previous publications by the authors^3,4^. These findings indicated the predominant presence of hierarchical hcp $\alpha ^\prime$ martensite laths under rapid cooling rates (2.56 $\times$ $10^7$– 4.82 $\times$ $10^4$  K/s). Moreover, the results indicated the evolution of anisotropic crystallographic texture due to steeper thermal gradients (1.48 $\times$ $10^6$– 1.58 $\times$ $10^7$ K/m) in the AM process. In order to understand the orientation-dependent elastic behavior, the printed block was subjected to ultrasonic scanning on four faces in the sets of XZ and YZ planes and compared against the reference wrought Ti6Al4V sample. The shear wave velocity (C$_T$) remained nearly same (3322 $\pm$ 20.12 m/s) for the wave plane (plane of vibration and direction of wave propagation) oriented at angles of 0$^\circ$ and 22.5$^\circ$ with respect to the build plane (XY) (Fig.  2a). Interestingly, there was a marked drop in the shear wave velocity (3245 $\pm$ 22.16 m/s) when the wave plane was aligned at 45$^\circ$ (Fig. 2a). At the wave plane orientation of 67.5$^\circ$, the shear wave velocity increased (3330 ± 19.45 m/s) back to nearly similar values as that at 0$^\circ$ and 22.5$^\circ$ orientations. Subsequently, again a drop in the velocity (3240 $\pm$ 21.01 m/s) nearly equal to that at 45$^\circ$ was measured at 90$^\circ$ orientation (Fig. 2a).

Contrary to the above measurements, the shear wave velocity nearly remained constant (3206 $\pm$ 10.12 m/s) for all the angular orientations in the reference wrought Ti6Al4V sample (Fig. 2b). As the density measurement via the EBME technique ($\rho$ = Z/c) marginally varied (LPBF-AM Ti6Al4V-4390 $\pm$ 60 kg/m $^3$ and Wrought Ti6Al4V-4680 $\pm$ 30 kg/m$^3$), the dynamic shear modulus calculated using Eq. (3) followed the trend similar to shear wave velocity as function of angular orientation in both LPBF-AM and wrought Ti6Al4V samples (Fig. 2c). Additional shear modulus contour maps corresponding to different shear wave plane orientation scanning on YZ plane of the LPBF-AM Ti6Al4V are presented in Fig. 2d. Based on the color legend, the significant variation in shear modulus can be noticed at 45$^\circ$ and 90$^\circ$ orientations of shear wave plane. Here it is noteworthy that under the set of LPBF-AM processing parameters employed in the present work, apparently, the three-dimensional defects such as cracks and porosity were not generated as they were neither detected by EBME technique nor by electron microscopy observations thereby indicating a marginal variation in the average density 4390 $\pm$ 60 kg/m$^3$. The dynamic bulk modulus, K$_d$ (K$_d$ = Zc) was estimated using the data generated through EBME tests. With these orientation-dependent dynamic shear modulus and the dynamic bulk modulus, other elastic constants such as dynamic Young’s modulus and dynamic Poisson’s ratio for LPBF-AM Ti6Al4V were calculated using elastic constants relations and are presented in Table 1. These observations suggested that there was a preferential crystallographic arrangement with respect to the build direction, causing a characteristic variation in the shear wave velocity within a LPBF-AM Ti6Al4V. It has been reported that shear waves transmitted through the samples can attenuate due to the presence of lattice discontinuities such as grain and phase boundaries^12^. Moreover, crystallographic texture can have a strong influence on the shear wave velocity depending on the orientation of crystallographic planes with respect to direction of wave propagation and vibration. With this perspective, the LPBF-AM Ti6Al4V samples were sectioned along with the faces (YZ and XZ) for microscopy observations using SEM/EBSD techniques.

As the laser beam scanning pattern was rotated through 90$^\circ$ in each subsequent layer, the microstructure and crystallographic texture in average remained consistent on the side face (XZ and YZ) of the cubical block. The representative SEM micrograph captured from the YZ plane of LPBF-AM Ti6Al4V cube is presented in Fig. 3a, which revealed the martensitic laths ($\alpha ^\prime$) within a prior columnar $\beta$ grains oriented along the build direction Z. The high magnification SEM micrograph as an inset of Fig. 3a indicates that these martensitic laths are extensively internally twinned. Also, the EBSD inverse pole figure (IPF) map generated from the same YZ plane of LPBF-AM Ti6Al4V cube consistently revealed the columnar prior $\beta$ grains laden with martensitic laths (Fig.  3b). Corresponding and additional 0001 pole figure texture plots generated from the other region of the YZ plane of LPBF-AM Ti6Al4V cube are presented in Fig. 3c and 3d. The reconstructed prior $\beta$ grain structure mainly revealed the columnar grain morphology with major fraction of <$001$> texture as presented in Fig.  3e,f. The length of the martensitic laths as measured from the IPF maps varied in the range of 20–60 $\upmu$m while their width remained between 1–4 $\upmu$m range. In addition, the physical orientation of martensitic laths was consistently at $\sim$ 45$^\circ$ or 90$^\circ$ to the prior $\beta$ boundaries (Fig. 3a,b). The pole figure texture plots corresponding to the HCP martensitic phase ($\alpha ^\prime$) indicated predominant orientation along <0001>, <11$\bar{2}$0>, and <10$\bar{1}$0> directions in different regions of YZ plane of LPBF-AM Ti6Al4V cube (Fig. 3c,d) suggesting that basal plane {0001}, primary {10$\bar{1}$0} and secondary prismatic {11$\bar{2}$0} planes are primarily parallel to the YZ plane of the LPBF-AM Ti6Al4V cube. In addition, the nature of internal twins within the martensite was investigated with high resolution SEM-EBSD and TEM analyses as presented in Fig. 4. The inverse pole figure map of single internally twinned martensite (Fig. 4a) was selectively analyzed for its matrix (Fig. 4b) and twinned region (Fig. 4c). Corresponding pole figures along {0001} (Fig. 4d) and {10$\bar{1}$1} (Fig. 4e) exhibited common {10$\bar{1}$1} pole (marked by open circles) in the twinned and the matrix region of the same martensite plate. Further analysis using TEM also confirmed the presence of internal twins as presented in the bright-field TEM image of Fig. 4f and corresponding electron diffraction pattern recorded a common [10$\bar{1}$1] type zone for both twin and matrix orientation (Fig. 4g). These commonly found 10$\bar{1}$1 type of twins in the LPBF-AM Ti6Al4V can be related to the transformation induced twins to accommodate the invariant plane strain due to the extremely rapid thermokinetic associated with LPBF-AM technique^3,4^. However, as the severe thermal stresses are also likely to be generated during subsequent multiple thermal cycles due to multi-track and multi-layer LPBF AM process, there is also a reasonable possibility of evolution of thermal deformation induced twins. A detailed investigation of the nature and evolution of such twins in LPBF-AM Ti6Al4V under multiple thermal cycles is undergoing and will be separately reported in due course of time.

The average elastic stiffnesses in different orientations (along and perpendicular to the build directions) were estimated from the polycrystalline IPF maps using the TSL-OIM analysis 8.0 software, which considers the single crystal data. Using multiple plot of IPF maps (5), the average elastic stiffness obtained for uniaxial tension along the build direction (Z) was 81.56 ± 10.62 GPa, whereas the average elastic elastic stiffness perpendicular (Y) to the build direction was 102.91 ± 8.01 GPa. These estimations follow the trend similar to that of experimentally observed variation in shear modulus (Fig. 2c), where the shear modulus drops at 90$^\circ$ orientation (Z) of shear wave plane to the build plane. With these observations, attempts are made to further analyze and propose rationale for the observed bulk elastic anisotropy in LPBF-AM Ti6Al4V.

The extent of scattering of the ultrasound shear wave is dependent on the grain size of the material, suggesting how far the crystallographic defects are spaced compared to its wavelength. In LPBF-AM Ti6Al4V, the width of columnar prior $\beta$ grains varied in the range of 30–80 $\upmu$m, while the martensitic laths possessed the dimensions of 1–4 $\upmu$m (width) and 20–60 $\upmu$m (length) (Fig. 3a,b). These final dimensions of the martensitic laths observed in the microstructure are the result of multiple thermal cycle experienced by each region of the printed component due to the multi-track and multi-layer nature of LBPF-AM technique. The multiple thermal cycle experienced by each region in LPBF-AM Ti6Al4V may have coarsen the martensite dimensions in each of the subsequent thermal cycles. In addition, internal twins with coherent boundaries within the martensitic laths are spaced at nanoscale dimensions (Figs. 3a, 4)^4^. Thus, an ultrasound shear wave of wavelength of $\sim$170 $\upmu$m (estimated based on the velocity of the shear wave in Ti6Al4V) is likely to undergo multiple types of scatterings as it encounters these crystallographic defects at different intervals. These scatterings may involve Rayleigh scattering (occurs when $\lambda$ grain size), stochastic scattering (occurs when $\lambda$ $\sim$ grain size), and diffusive scattering (occurs when $\lambda<$ grain size)^15^. Furthermore, reportedly high LPBF-AM process-inherent defect density^4^ is likely to scatter the shear wave and attenuate its velocity. However, as these defects are ubiquitous in the LPBF-AM material, they are unlikely to have directional effect in the attenuation of shear wave velocity. Therefore, the attenuation at 45$^\circ$ and 90$^\circ$ is likely to be a cause of crystallographic bulk texture.

During fabrication of Ti6Al4V block via LPBF-AM technique, rapid thermokinetics yield steeper thermal gradients in the range of 1.48 $\times$ 10$^6$ K/m to 1.58 $\times$ 10$^7$ K/m along the build direction, which were computationally predicted in the earlier reports of authors^3,4^. Under such steeper thermal gradients, the $\beta$ grains heterogeneously nucleate from the substrate or previously printed layer and competitively grow along <$001$>, which wields high thermal conductivity in cubic systems producing a strong 001 texture (Fig.  3e,f). This has also been confirmed in several studies, which have traced the prior $\beta$ texture based on the Burgers orientation relationship of $\beta$ and $\alpha ^\prime$^26–28^. The schematic of such prior $\beta$ grains depicting BCC unit cell orientation along <$001$> directions is presented in Fig. 5a. Here, we have assumed that BCC unit cell in a given $\beta$ grain is directed along [001] direction parallel to build direction (Z). When the martensitic start temperature is reached, the BCC unit cell of $\beta$ transforms to martensitic ($\alpha ^\prime$) HCP unit cell. During this displacive transformation, a single BCC unit cell can select one of the 12 possible variants of $\alpha ^\prime$ as per Burgers orientation relationship of $\{0001\} _{\alpha ^\prime } || \{110\}_{\beta };<1120>_{\alpha ^\prime }||<111>_{\beta }$^28,29^. These twelve (6$\times$2) possible equivalent orientation variants of $\alpha ^\prime$ are allowed as there are 6 possible $\{110\}_{\beta }$ planes and two distinct combinations of parallel directions $<1120>_{\alpha ^\prime } || <111>_{\beta }$ on (0001)$_{\alpha ^\prime }$ basal plane. Considering BCC unit cell oriented along [001] || build direction (Z), its possible 6 {110}$_{\beta }$ planes forming basal {0001}$_{\alpha ^\prime }$ plane and giving rise to HCP unit cell are depicted in Fig. 5b–g. Here, it can be noticed that $\alpha ^\prime$ variants formed along (110) and (1$\bar{1}$0) planes have [0001] direction perpendicular to prior $\beta$ [001] direction (Fig. 5b,c). On the contrary, other variants of $\alpha ^\prime$ formed on (01$\bar{1}$), (011), (101), and ($\bar{1}$01) planes have their [0001] direction at ± 45$^\circ$ to prior $\beta$ [001] direction (Fig. 5d–g). Therefore, out of 12 variant possibilities, 8 variants of $\alpha ^\prime$ HCP have their c-axis inclined at ± 45$^\circ$ to the build direction, and the remaining 4 variants have their c-axis perpendicular to the build direction. This rationalizes the morphology of martensite laths within the prior $\beta$ grains oriented specifically at $\sim$ 45$^\circ$ or 90$^\circ$ to the columnar boundaries. The 0001 pole figure texture plots pointed earlier also supports the Burgers orientation relationship with 12 variants, which allows primary and secondary prismatic planes, and basal planes of HCP to align parallel to the YZ plane of the Ti6Al4V cube (Figs. 3c,d,  5b–g).

In HCP Ti, the lowest elastic stiffness modulus corresponds to C$_{44}$ (46.5 GPa), which is equivalent to the shear modulus corresponding to the several shear plane and direction systems in HCP crystal^30,31^. These systems involve primary prismatic planes {01$\bar{1}$0} with <$0001$> directions, secondary prismatic planes {12$\bar{1}$0} with <$01\bar{1}0$> directions and the basal plane {0001} sheared along <$01\bar{1}0$> directions^30,31^. The directions are usually referred as elastically soft direction which have low linear atomic density along them, whereas high linear atomic density directions are elastically hard directions. In Titanium, due to c/a ratio of 1.58, <$0001$> is a hard (G$_{<0001>}$ = 46.5 GPa) directions while $<01\bar{1}0>$ directions are soft (G$_{<0110>}$ = 39.9 GPa) directions^30^. Soft directions due to low atomic density tend to attenuate the shear vibrations relayed to the neighboring atoms, thereby reducing the resultant shear velocity and, in turn, shear modulus. In the present study, the direction of shear wave propagation was unchanged along X-axis (Fig. 1c), whereas the direction of shear wave vibration was changed (by rotation), thereby changing the shear wave plane. At the 45$^\circ$ orientation of the shear wave plane, the direction of shear vibration coincides with $<10\bar{1}0>$ directions for the $\alpha ^\prime$ variants corresponding to (01$\bar{1}$), (011), (101), and (10$\bar{1}$) planes of prior β phase (Fig. 5d–g). Hence, the velocity of shear wave is likely to reduce while vibrating along these directions. When the shear wave plane is oriented at 90$^\circ$ to the build plane, the direction of vibration nearly coincides with $<10\bar{1}2>$ directions of $\alpha ^\prime$ variants corresponding to (01$\bar{1}$), (011), (101), and (10$\bar{1}$) planes of prior $\beta$ phase, which is another soft direction (G$_{<1012>}$ = 41.7 GPa) in HCP-Ti^30^. In addition, for these variants (Fig.  5d–g), it can be noticed that $<10\bar{1}2>$ corresponds to the $<001>_{\beta }$, which is lowest linear atomic density and thereby elastically soft direction in BCC. The schematic visualization of shear wave plane coinciding with the elastically soft directions is depicted in Fig. 6a,b. At 45$^\circ$ orientation of the shear wave plane with respect to the build plane (XY), the direction of shear vibration coincides with [01$\bar{1}$0] for the $\alpha ^\prime$ variant transformed on (011)$_{\beta }$ plane (Fig.  6a). Similarly, the rest of $\alpha ^\prime$ variants having their c axis aligned at ± 45$^\circ$ to the prior [001]$_{\beta }$ direction are likely to have the shear wave plane (with 45$^\circ$ orientation) coinciding on $<01\bar{1}0>$ direction. Along the same lines, with 90$^\circ$ orientation of the shear wave plane with respect to the build plane (XY), the vibrating direction coincides with $<10\bar{1}2>$ directions (Fig. 6b). Therefore, in spite of 12 different possibilities of variants of $\alpha ^\prime$ formed within the strongly textured prior $\beta$ matrix, the family of elastically soft directions has maximum probability to coincide with the direction of shear vibration at 45$^\circ$ and 90$^\circ$ orientations passing through the bulk of the printed component. This reflects the bulk crystallographic texture and corresponding orientation dependence of dynamic elastic moduli of LPBF-AM Ti6Al4V.

## Conclusion

In the current study, texture driven elastic response of additively manufactured Ti6Al4V alloy using laser powder bed fusion technique was investigated using integrated EBME and shear wave velocity measurement approach. Change in the bulk elastic stiffness at shear wave plane oriented at 45$^\circ$ and 90$^\circ$ with respect to the plane normal to the build direction was recognized due to a drop in the shear wave velocity at these orientations. SEM microstructures and EBSD IPF maps revealed the physical morphology of martensite laths oriented at 45$^\circ$ and 90$^\circ$ to the columnar $\beta$ grain boundary. The 0001 pole figures identified the texture mainly in $<0001>,<11\bar{2}0>, <10\bar{1}0>$ directions in different regions of YZ plane of LPBF-AM Ti6Al4V cube. This assisted the proposed rationale based on the orientation variants of the product martensite phase with respect to the parent $\beta$ phase, which increases the probability of aligning the soft directions to the shear vibration direction.
